# A Case of Amiodarone-Induced Thyrotoxicosis Presenting With Methimazole-Induced Agranulocytosis

**DOI:** 10.7759/cureus.63858

**Published:** 2024-07-04

**Authors:** Neha Meda, Suhail Saad-Omer, Moises Matos, Mustafa Kinaan

**Affiliations:** 1 Internal Medicine, University of Central Florida Hospital Corporation of America (HCA) Healthcare Graduate Medical Education (GME), Orlando, USA; 2 Endocrinology, Miami Veterans Affairs Healthcare System, Miami, USA; 3 Endocrinology, Diabetes, and Metabolism, University of Central Florida Hospital Corporation of America (HCA) Healthcare Graduate Medical Education (GME), Orlando, USA

**Keywords:** interleukin-6 (il-6), thyrotoxicosis, amiodarone, methimazole, drug-induced agranulocytosis

## Abstract

Amiodarone is a class III anti-arrhythmic drug found to be effective in treating multiple life-threatening arrhythmias, including paroxysmal atrial fibrillation. Despite its effectiveness, amiodarone has been found to result in thyroid dysfunction. Amiodarone-induced thyrotoxicosis (AIT) is classified as type 1, which often develops in those with autoimmune hyperthyroid conditions, or type 2, which occurs because of destructive thyroiditis in an apparently normal thyroid. Differentiating between both types often poses a clinical and therapeutic dilemma, as AIT 1 is treated with thionamides, whereas AIT 2 requires steroids for treatment. We present a case of a patient with AIT who was treated empirically for both subtypes with methimazole and prednisone without clinical improvement. Methimazole was later stopped due to concern for agranulocytosis, and the patient was then treated with cholestyramine, metoprolol, and prednisone. Given persistent thyrotoxicosis, the decision was made to proceed with surgical intervention. The patient underwent a successful total thyroidectomy without complications. The patient‘s condition clinically improved post-surgery and was discharged home on post-operative day 2 in stable condition. Prednisone was tapered over two weeks, and he was started on a weight-based dose of levothyroxine. He continues to follow up in our clinic for postoperative hypothyroidism and is clinically and biochemically euthyroid.

## Introduction

Amiodarone is a class III anti-arrhythmic drug found to be effective in treating multiple life-threatening arrhythmias including paroxysmal atrial fibrillation. Despite its effectiveness, amiodarone has been found to result in thyroid dysfunction. Amiodarone-induced thyrotoxicosis (AIT) is classified as type 1 which often develops in those with autoimmune hyperthyroid condition or type 2 which occurs because of destructive thyroiditis in an apparently normal thyroid. Differentiating between both types often poses a clinical and therapeutic dilemma as AIT 1 is treated with thionamides, whereas AIT 2 requires steroids for treatment. We present a case of a patient with AIT who was treated empirically for both subtypes with methimazole and prednisone without clinical improvement. Methimazole was later stopped given concern for agranulocytosis, and the patient was then treated with cholestyramine, metoprolol, and prednisone. Given persistent thyrotoxicosis, the decision was made to proceed with surgical intervention. The patient underwent a successful total thyroidectomy without complications. The patient‘s condition clinically improved post-surgery and was discharged home on post-operative day 2 in stable condition. Prednisone was tapered over two weeks, and he was started on a weight-based dose of levothyroxine. He continues to follow up in our clinic for postoperative hypothyroidism and is clinically and biochemically euthyroid.

This case was presented at the American Association of Clinical Endocrinology (AACE) annual meeting in 2023 and published as an abstract in Endocrine Practice in May 2023 [1].

## Case presentation

A 67-year-old man presented to the hospital due to a two-week history of palpitations, elevated heart rate, and associated chest tightness. He reported mild tremors, diarrhea, and unintentional weight loss. His medical history was remarkable for hypertension, atrial fibrillation, and a prior cerebral vascular accident at the age of 40. The patient denied any personal or family history of thyroid disease. He was diagnosed with atrial fibrillation three years ago and treated with amiodarone for about two years. His initial examination was notable for an irregularly irregular heart rhythm, clear lungs to auscultation bilaterally, and a normal neck without jugular venous distension or goiter. His initial EKG on admission showed atrial fibrillation with rapid ventricular response and a heart rate of 120 bpm. Initial labs were notable for hyperthyroidism (Table 1).

**Table 1:** Lab results of thyroid hormones, thyroid antibodies, WBC count, and ANC from hospital admission WBC: white blood cell, ANC: absolute neutrophil count, TSH: thyroid-stimulating hormone

Test	Lab value	Normal range
TSH	<0.01 uIU/ml	0.36-3.74 uIU/ml
Free T4	3.32 ng/dL	0.76-1.75 ng/dL
Total T3	237 ng/dL	86-192 ng/dL
Thyrotropin receptor-blocking antibodies	<1.10 IU/mL	0.0-1.75 IU/mL
Thyroglobulin antibodies	<20 IU/mL	0.0-40.0 IU/mL
Thyroid peroxidase antibodies	<10.0 IU/mL	0.0-35I U/mL
WBC count	8200 cells/mL	4000-10000 cells/µL
ANC	4887 cells/mL	1780-5380 cells/mL

**Figure 1:**
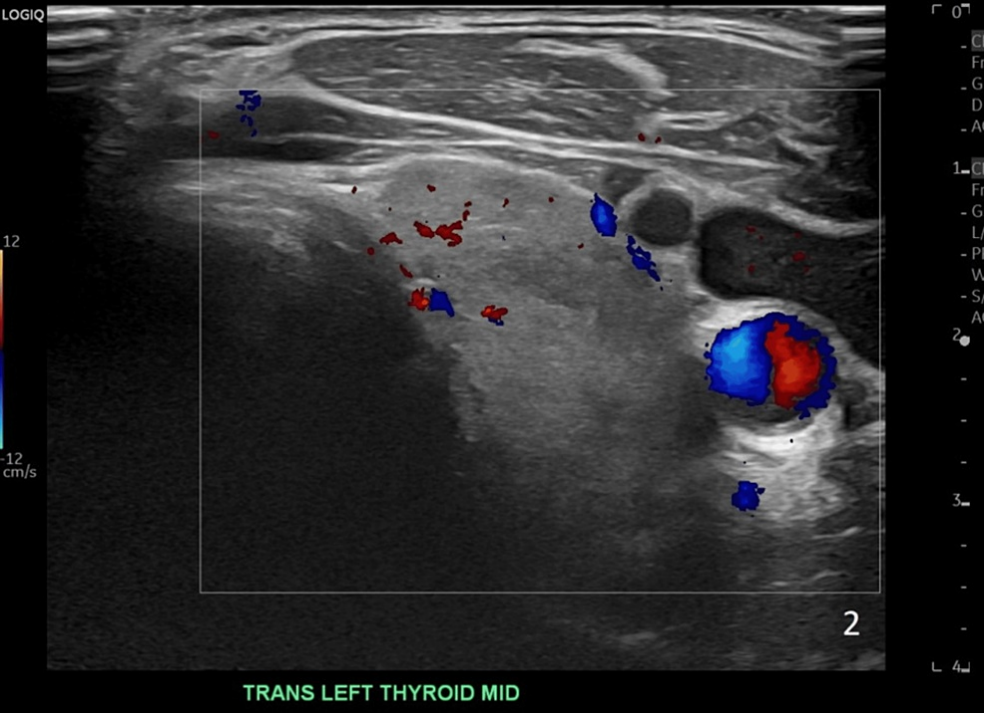
Image of the left lobe thyroid ultrasound

**Figure 2:**
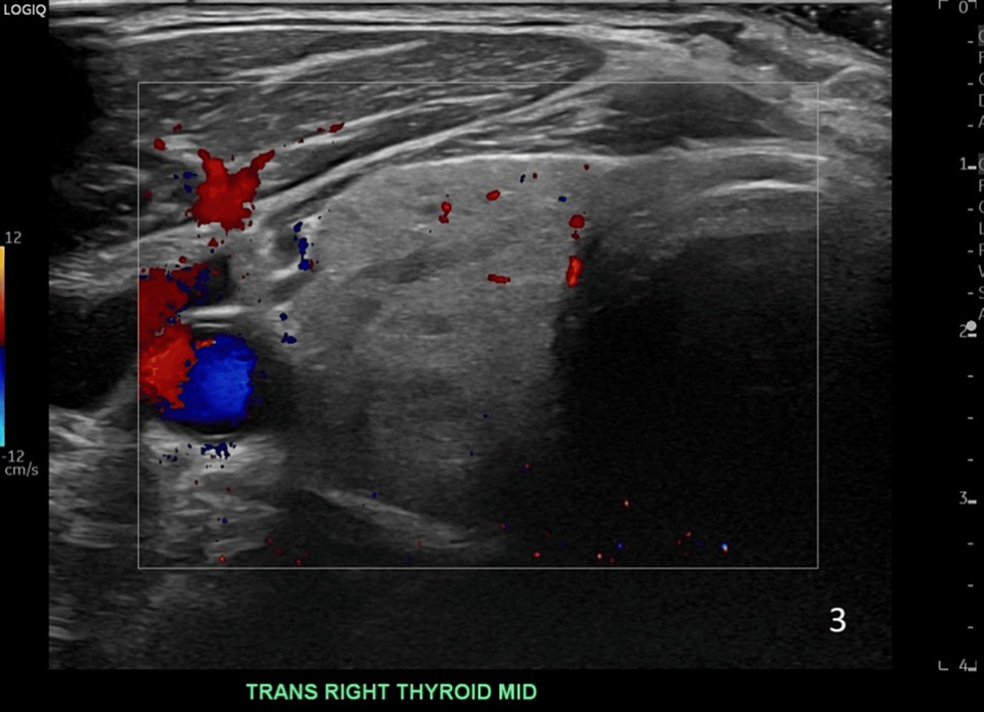
Image of the right lobe thyroid ultrasound

Thyroid ultrasound showed a slightly enlarged and heterogeneous thyroid without discrete nodules and normal vascularity (Figures 1-2). A preliminary diagnosis of AIT was made. He was treated empirically for both AIT 1 and AIT 2 with methimazole 40 mg and prednisone 40 mg daily.

He presented to the hospital approximately one month later with similar complaints. He was again noted to be in atrial fibrillation with a rapid ventricular response. His Burch-Wartofsky score on presentation was 30, which is suggestive of an impending thyroid storm. Initial labs during his second admission were notable for agranulocytosis and persistent hyperthyroidism, despite methimazole and prednisone therapy for over a month (Table 2).

**Table 2:** Lab results of thyroid hormones, WBC count, ANC count, and IL-6 from subsequent hospital admission WBC: white blood cells, ANC: absolute neutrophil count, IL-6: interleukin 6

Test	Day 1 of admission	Day 2 of admission
White blood cell count	4000 cells/mL	2600 cells/mL
Absolute neutrophil count	520c ells/mL	62 cells/mL
Thyroid-stimulating hormone	<0.01 uIU/mL	-
Free T4	4.01 ng/dL	-
Total T3	146 ng/dL	-
IL-6	6.6 pg/mL (0.0-13.0)	-

Methimazole was discontinued upon admission, and the patient was treated with cholestyramine, metoprolol, and prednisone. Given the persistent hyperthyroidism and the development of agranulocytosis, thyroidectomy was necessary. His agranulocytosis improved with the discontinuation of methimazole and resolved completely a day after administration of filgrastim 300 mcg. He was treated with potassium iodide (SSKI) in preparation for thyroidectomy to reduce vascularity and decrease intra-operative blood loss. Given his prolonged exposure to high-dose steroids for treatment of AIT, he was treated with hydrocortisone during the perioperative period. Early in the morning, cortisol was checked and found to be 7.35 mcg/dL. He was considered at risk for adrenal insufficiency from hypothalamic-pituitary axis suppression due to prolonged exposure to steroids. He underwent a successful total thyroidectomy without complications. He did well following his surgery and was discharged home in stable condition on postoperative day two. His prednisone was tapered off over two weeks, and he was started on a weight-based dose of levothyroxine. He continues to follow up in our endocrine clinic and is clinically and biochemically euthyroid.

## Discussion

Amiodarone is a class III anti-arrhythmic drug that has been found to be effective in the treatment of multiple life-threatening arrhythmias, including paroxysmal atrial fibrillation, as well as in recurrent severe ventricular arrhythmias [2]. Despite its effectiveness, it has been found to frequently induce thyroid dysfunction in as many as 36% of patients who take this medication chronically [3]. This side effect is thought to be due to either excessive iodine overload related to its biochemical structure or to its direct cytotoxicity on thyrocytes [4]. AIT was found to develop in 3% of patients in North America, while those living in iodine-depleted areas were found to have a higher incidence of 10% [5]. Bogazzi et al. helped to classify AIT into two major categories: type 1 and type 2 (Table 3) [6,7].

**Table 3:** Differences between AIT 1 and AIT 2 AIT: amiodarone-induced thyrotoxicosis, USG: ultrasound sonography, TSI: thyroid-stimulating immunoglobulin, TRAB: thyroid-stimulating hormone receptor antibody, IL-6: interleukin 6

	Type 1	Type 2
Mechanism	Excessive thyroid hormone production due to iodine overload	Destructive thyroiditis leads to release of preformed thyroid hormone into circulation
Pre-existing thyroid disease	Yes, patients may have latent autoimmune thyroid disease	No
Prevalence	Most common type in iodine-deficient regions	Most common type in the United States
Imaging	Normal or increased vascularity on USG	Decreased vascularity on USG
Labs	May have positive TSI, TRAB, no IL-6 elevation, normal/high thyroglobulin	No detectable thyroid antibodies, may have IL-6 elevation, low thyroglobulin
Treatment	Antithyroid medications	Steroids

AIT 1 typically occurs in patients with an underlying thyroid pathology such as Graves’ disease or multinodular goiter. In these patients, iodine overload secondary to amiodarone leads to follicular hyperreactivity. However, in AIT 2, thyrotoxicosis results from the cytotoxic effect of amiodarone and results in destructive thyroiditis, causing excess release of T3 and T4 into the circulation [6]. The effect of amiodarone may vary, taking several weeks to several years to manifest in some patients [3]. The majority of cases in North America are AIT 2, whereas AIT 1 predominates in iodine-deficient areas of the world [5]. The differentiation of these two types of AIT often poses a unique challenge for clinicians, as some patients may have a mixture of both mechanisms. Nevertheless, determining which type of AIT a patient has is imperative in deciding subsequent management and treatment. Patients with AIT may have typical hyperthyroid symptoms, including palpitations, tremors, and sweating, or be completely asymptomatic, likely due to the beta-adrenergic blockade of amiodarone [8]. This is why a thoroughly taken history and conducted physical examination are imperative to help determine whether the patient has a pre-existing thyroid condition. A physical exam may reveal a goiter or exophthalmos. Ultrasounds of the thyroid may show an enlarged gland or nodular goiter. Thyroid peroxidase may also be present. These features may sway the differential in favor of AIT 1. Inflammatory markers such as IL-6 may be elevated in AIT 2, indicating ongoing thyroiditis; however, some patients with AIT 2 have been found to have low IL-6 levels, likely due to the assay being used, limiting its overall usefulness [9]. One tool that is gaining favor to help in the rapid and early diagnosis of AIT is color flow Doppler sonography (CFDS). This test helps shed light on the morphology of the thyroid by determining the amount of blood flow within the organ. Several recent studies have shown that the absence of hyperflow on CFDS is suggestive of AIT 2, while an increase in vascularity supports a diagnosis of AIT 1 [10].

The treatment of AIT is often very challenging for the clinician, especially in cases where the type is uncertain. The first issue that must be addressed is the continuation or discontinuation of amiodarone. Patients with severe, resistant tachyarrhythmias would require amiodarone to be continued regardless of the risk of AIT aggravation or reoccurrence [6]. Some experts believe that due to amiodarone’s prolonged half-life of six months secondary to its lipo-solubility, continuing or discontinuing the drug would not influence AIT management [6]. Nevertheless, multiple studies have shown amiodarone continuation increased the rate of reoccurrence in patients with AIT 2 and also delayed the response of treatment to glucocorticoids and thionamides [11]. ATA recommends taking into consideration the cardiologist’s decision on whether an alternative to amiodarone is available [12].

AIT 1 is typically treated with antithyroid thionamides at high doses in order to overcome high iodine concentrations. Thionamides are typically continued for about three to six months until euthyroidism is restored [13]. Potassium perchlorate may also be started in the first few weeks of treatment to decrease thyroid uptake of iodine and make the thyroid more sensitive to the antithyroid medication (ATD); however, due to its toxicity and risk of aplastic anemia, its use is limited to short periods of time [14]. AIT 2 is usually self-limited and, in mild cases, can resolve spontaneously in about 20% of cases. However, due to the increased mortality associated with this condition in cardiac patients, treatment is typically started [15].

AIT 2 is treated with glucocorticoids for anti-inflammatory effects. Prednisone is started at 40 mg daily and tapered over two to three months [15]. In cases where AIT is mixed or unidentified, treatment should typically begin with ATD and oral corticosteroids [7]. A large cohort study revealed that the prevalence and incidence of ATD-AGRAN were higher in AIT patients compared with non-AIT patients [16]. Total thyroidectomy is usually considered under several circumstances, the most common being insufficient response to drug treatment with ATD and corticosteroids, with other indications being rapid deterioration of cardiac function as well as advanced heart disease and malignant arrhythmias [17]. In a recent observational study with 207 AIT patients, those who underwent thyroidectomy showed lower mortality and significant improvement in LVEF than those who were only treated medically, especially in patients with LVEF <40% [18]. Another smaller retrospective cohort study at Mayo Clinic revealed similar findings supporting the fact that thyroidectomy does help to rapidly resolve thyrotoxicosis and improve cardiac function, but that complications of the procedure in AIT were higher than those with other thyrotoxic etiologies [19]. Finally, plasmapheresis remains another interesting treatment modality that has shown promise, but so far evidence has only been limited to case reports [20].

## Conclusions

This is a unique case of AIT that could not be classified into either type 1 or type 2 and was treated empirically for both types. These uncategorized cases of AIT are difficult to treat with medical therapy and likely require surgical management, as seen in this case. Further research is warranted into treatment and non-surgical options for treating AIT, along with guidelines on the treatment of uncategorized or mixed AIT.
